# A pilot randomized controlled trial of pioglitazone for the treatment of poorly controlled asthma in obesity

**DOI:** 10.1186/s12931-015-0303-6

**Published:** 2015-11-26

**Authors:** Anne E. Dixon, Meenakumari Subramanian, Michael DeSarno, Kendall Black, Lisa Lane, Fernando Holguin

**Affiliations:** University of Vermont, Burlington, VT USA; University of Pittsburgh, Pittsburgh, PA USA; Division of Pulmonary and Critical Care Medicine, Given D209, 149 Beaumont Avenue, Burlington, VT 05405 USA

**Keywords:** Obesity, Asthma, Thiazolidinedione, Asthma control

## Abstract

**Background:**

Obese asthmatics tend to have poorly controlled asthma, and resistance to standard asthma controller medications. The purpose of this study was to determine the efficacy of pioglitazone, an anti-diabetic medication which can alter circulating adipokines and have direct effects on asthmatic inflammation, in the treatment of asthma in obesity.

**Methods:**

A two-center, 12-week, randomized, placebo-controlled, double-blinded trial. Treatments were randomly assigned with concealment of allocation. The primary outcome was difference in change in airway reactivity between participants assigned to pioglitazone versus placebo at 12 weeks.

**Results:**

Twenty-three participants were randomized to treatment, 19 completed the study. Median airway reactivity, measured by PC_20_ to methacholine was 1.99 (IQR 3.08) and 1.60 (5.91) mg/ml in placebo and pioglitazone group at baseline, and 2.37 (15.22) and 5.08 (7.42) mg/ml after 12 weeks, *p* = 0.38. There was no difference in exhaled nitric oxide, asthma control or lung function between treatment groups over the 12 week trial. Participants assigned to pioglitazone gained a significant amount more weight than those assigned to placebo (pioglitazone group mean weight 113.6, CI 94.5-132.7 kg at randomization and 115.9, CI 96.9-135.1 at 12 weeks; placebo mean weight 127.5, CI 108.4 – 146.6 kg at randomization and 124.5, CI 105.4 – 143.6 kg at 12 weeks; *p* = 0.04).

**Conclusions:**

This pilot study suggests limited efficacy for pioglitazone in the treatment of poorly controlled asthma in obesity, and also the potential for harm, given the weight gain in those assigned to active treatment, and the association between increased weight and worse outcomes in asthma.

**Trial Registration:**

Clinicaltrials.gov (NCT00634036)

## Background

The majority of adults with poorly controlled asthma in the United States are obese [1]. Obese asthmatics are more likely to have moderate persistent or severe asthma and suffer from more frequent exacerbations [2]. One study suggested that obese asthmatics had a nearly five-fold increased risk of hospitalization for asthma compared with lean asthmatics [3]. One reason for this poor asthma control, is that they do not respond as well to standard asthma therapy [4, 5], and *in vitro* studies suggest that they have intrinsic steroid resistance [6]. There is an urgent need to find effective treatments for obese asthmatics, these patients now represent the majority of asthmatics, and are more likely to have poorly controlled disease than lean individuals [1].

The pathogenesis of asthma in obesity is likely very different from the pathogenesis of asthma in lean individuals with typical early-onset allergic asthma. The mass of adipose tissue in obesity may contribute to mechanical changes in the respiratory system. In our earlier work we have shown that obese asthmatics have evidence of increased airway oxidative stress [7, 8], and decreased peripheral lung compliance [9]. The causes of this increased airway oxidative stress and decreased peripheral lung compliance are not known, but could be related to mediators produced by adipose tissue which may have direct effects on the lung. In obese individuals adipose is an active metabolic organ which produces multiple mediators such as leptin which have widespread pro-inflammatory effects, and also decreased levels of mediators with anti-inflammatory effects such as adiponectin [10]. Indeed, we have shown that obese asthmatics have increased leptin and decreased adiponectin in visceral adipose tissue compared to obese non-asthmatics, and that visceral fat leptin expression correlates directly with airway reactivity in obese asthmatics [11]. Increased leptin and decreased adiponectin from adipose tissue may contribute to the pathogenesis of asthma in obesity.

Certain medications alter the production of adipokines by adipose tissue. The thiazolidinediones (TZDs) are a class of drugs that activate the nuclear receptor peroxisome proliferator activated receptor gamma (PPARγ), a transcription factor for genes involved in lipid biosynthesis and glucose metabolism. These medications are primarily used in the treatment of diabetes. They are insulin sensitizing drugs which act by increasing glucose uptake by adipose tissue and muscle, and also by altering adipokine expression; because of their mechanism of action, they do not produce hypoglycemia in non-diabetic subjects. Previous studies have suggested that treatment with TZD’s are not only efficacious in diabetes, but can be effective in the treatment of metabolic complications of obesity such as steatohepatitis [12]. TZD’s could be efficacious in the treatment of obese asthma through metabolic effects on adipose tissue.

PPARγ agonists also have direct effects on the airway in asthma. PPARγ is expressed in bronchial epithelium and submucosa of human (non-obese) asthmatic subjects [13]. Data from animal experiments suggest that treatment with PPAR-γ agonists reduce the ability of antigen presenting cells to activate T cells and induce eosinophilic airway inflammation – inflammation characteristic of T_H_2 high asthma [14]. PPARγ agonists reduce airway hyperresponsiveness, measures of oxidative stress, collagen deposition and mucus hypersecretion in an allergic mouse models of asthm [15–17]. TZD medications could be efficacious in the treatment of asthma through direct effects on the airway.

We hypothesized that in obese poorly controlled asthmatics, the addition of pioglitazone to standard asthma therapy would reduce bronchial hyperresponsiveness when compared to placebo. We also hypothesized that pioglitazone would improve asthma control and lung function compared with placebo.

## Methods

### Study Design

A randomized, placebo-controlled, parallel arm, double-blinded study of pioglitazone for the treatment of poorly controlled obese asthmatics. This study was conducted at two centers (the Universities of Vermont and Pittsburgh) from March 2010 – April 2013. After a four week run-in, participants were randomized to pioglitazone starting at 30 mg/day and increased to 45 mg/day after two weeks, or matching placebo (provided by Takeda). Allocation ratio was 1:1. An independent 3rd party (Investigational Drug Services at the University of Pittsburgh) prepared and distributed blinded study drug kits to the study sites. The University of Pittsburgh Investigational Drug Service maintained the master randomization schema and communicated all study drug assignments to the clinical sites when patients were enrolled. Block randomization was performed to assure a balanced distribution of covariates (age and gender). The blind was broken for the data analysis, after completion of all study procedures. This trial was registered at clinicaltrials.gov (NCT00634036), an Investigational New Drug Application was approved by the FDA, and the study was approved by the institutional review board at both institutions. Informed consent was obtained from all study participants.

### Eligibility Criteria

The goal of this study was to enroll obese adults with moderate to severe asthma. Inclusion criteria included: physician diagnosis of asthma, poorly-controlled asthma defined as a Juniper asthma control questionnaire (ACQ) score ≥ 1.5 [18], absence of active smoking (none in last 12 months and <10 pack-years total), BMI 30–60 kg/m^2^, ages 18–60 years, PC_20_ to methacholine of < 16 mg/ml, ability to understand and sign the consent form, on a stable dose of inhaled corticosteroid for at least 4 weeks prior to study entry, FEV_1_ ≥ 60 % predicted. Exclusion criteria included the following: use of systemic steroids within the past 4 weeks; lung disease other than asthma; significant non-pulmonary co-morbidities; B-type natriuretic peptide (BNP) >400 pg/mL; pregnant or lactating; currently taking a beta blocker, a CYP2C8 inhibitor or inducer such as gemfibrozil or rifampin, a thiazolidinedione, or allergic to TZD; use of nutritional supplements, antioxidants, or multivitamins; illicit drug use within the past year; current or active upper respiratory infection; asthma exacerbation within the past 4 weeks; institution of treatment for sleep apnea planned; clinically significant abnormalities on 12-lead electrocardiogram.

### Outcomes

The primary outcome measure was the change in airway methacholine reactivity at 12 weeks compared with baseline. Secondary outcomes included changes in Juniper Asthma Control Score, spirometry and exhaled nitric oxide, all measured at randomization, week 2, week 6 and week 12.

Prior to randomization, renal and liver function tests, a complete blood count, brain natriuretic peptide (BNP) and a fasting glucose were obtained, and these were rechecked at 2, 6 and 12 weeks to monitor for any adverse effects of Pioglitazone.

### Statistical analysis

In order to test for significant treatment effects on outcome measures of interest, mixed model repeated measures analyses of variance were conducted. In these models, the outcome variables were treated continuously; variables that were determined to be non-normally distributed (airway reactivity to methacholine, for example) were log-transformed in order to satisfy the normality assumption of the model. Fixed effects used in the models were visit and treatment group, with random effect of subject. The group by visit interaction results of the analyses were used to test for significant differences between groups in changes in outcome measures over time, using significance level alpha = 0.05. Analyses were done using SAS, version 9.2, statistical analysis software (SAS Institute, Inc., Cary, NC, USA).

### Sample Size

The original sample size calculation was based upon estimates of clinically meaningful mean differences and standard deviations derived from our studies of obese asthmatics. The analysis was based upon a repeated measures analysis of variance approach. The primary outcome was airway reactivity to methacholine. We assumed that after treatment with Pioglitazone, a 20 % fall in FEV_1_ be detected using a mean methacholine dose of 6 mg/ml, an improvement over the baseline estimate of 3 mg/ml. It was assumed that those in the control group would show no improvement over baseline. Using a within-person standard deviation of 3.0, and the assumptions indicated above, 17 individuals per group would provide 80 % power with a 5 % significance level to detect a difference between patients assigned to active treatment versus placebo. Due to new safety concerns which emerged about pioglitazone during the trial affecting patient recruitment, a decision was made to halt the study early after recruitment of only 23 subjects.

## Results

**Fig. 1:**
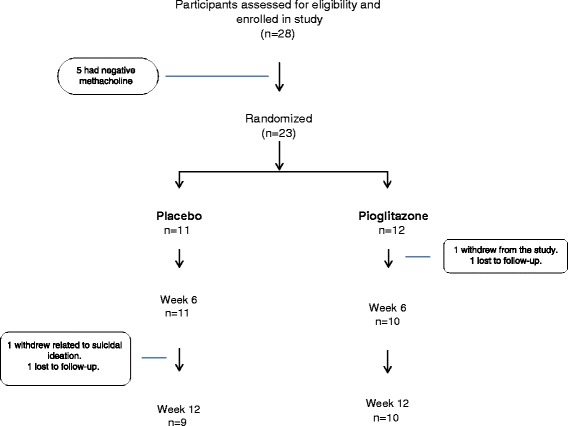
Eligibility screening, randomization and follow up of study participants. All patients were included in the intention to treat analysis based upon the assigned treatment

The numbers of patients enrolled, randomized and completing the study are shown in  Figure 1. The baseline demographics of the participants are shown in Table 1. The majority of study subjects were female and Caucasian. They reported a high prevalence of comorbidities such as atopy, GERD and depression. They had poor asthma control confirmed with a high proportion experiencing a self-reported asthma exacerbation in the last 12 months, and oral corticosteroid use in the prior 12 months. Asthma was also poorly controlled as measured by the Juniper Asthma Control Score (with a score of 1.5 or greater being indicative of poor asthma control).

**Table 1:** Baseline demographics of the study population

	Placebo	Pioglitazone
n	11	12
Female (%)	8 (72)	7 (52)
Age	41 ± 14.1	39.4 ± 10
Age asthma onset	21 ± 18.9	15 ± 11
Race		
Caucasian	9 (82)	8 (66.6)
Black	2 (18)	4 (33.3)
BMI	43.5 ± 7.8	38.8 ± 6.8
Seasonal allergies (%)	10 (91)	10 (83)
GERD (%)	5 (45)	7 (58)
Depression (%)	6 (55)	7 (58)
Asthma exacerbation in last 12 months (%)	7 (64)	6 (50)
Oral steroids in last 12 months (%)	4 (36)	7 (58)
Inhaled corticosteroid n (%)	11 (100)	12 (100)
High dose* (n)	5	4
Medium dose (n)	4	8
Low dose (n)	2	0
Long acting beta agonist, n (%)	9 (81.8)	10 (83.3)
Short acting beta agonist,n (%)	11 (100)	11 (92)
FeNO (ppb)	30.8 ± 28.4	27.6 ± 27.8
IgE (IU/ml)	575 ± 544.7	291.2 ± 313.2
Juniper Asthma Control	2.48 ± 1.30	1.75 ± 0.63
FEV1 (% pred pre BD)	81.5 ± 15.2	82.3 ± 12.1
FVC (% pred. pre BD)	85.3 ± 16.2	86.8 ± 13.7
FEV1/FVC (% pred. pre BD)	94.9 ± 7.67	95.3 ± 11.12
FEV1 (% pred. post BD)	88.1 ± 4.6	77.3 ± 25.1
FVC (% pred. post BD)	88.3 ± 16.3	79.5 ± 27.6
FEV1/FVC (% pred. post BD)	97.8 ± 7.82	90.9 ± 31.2

*High, medium and low dose of ICS defined according to GINA guidelines [41]

**Fig 2:**
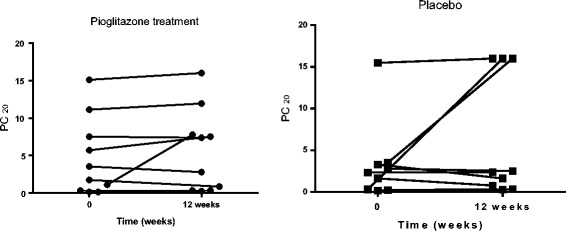
Change in airway reactivity in participants assigned to placebo versus pioglitazone (*p* = 0.38)

Participants had a similar level of airway reactivity at baseline. There was no significant difference in the change in airway reactivity in participants assigned to pioglitazone versus placebo during the treatment phase of the trial (Table 2, Fig. 2).

**Table 2:** Change in airway reactivity

	placebo	pioglitazone	*P*-value*
PC_20_ at baseline (mg/ml)	1.99 (3.08)	1.60 (5.91)	
	*n* = 11	*n* = 12	
PC_20_ at 12 weeks (mg/ml)	2.37 (15.22)	5.08 (7.42)	
	*n* = 9	*n* = 10	0.38

Values shown are median and IQR

*P-value shown for mixed model repeated measures analysis of variance, treatment by time interaction

**Fig 3:**
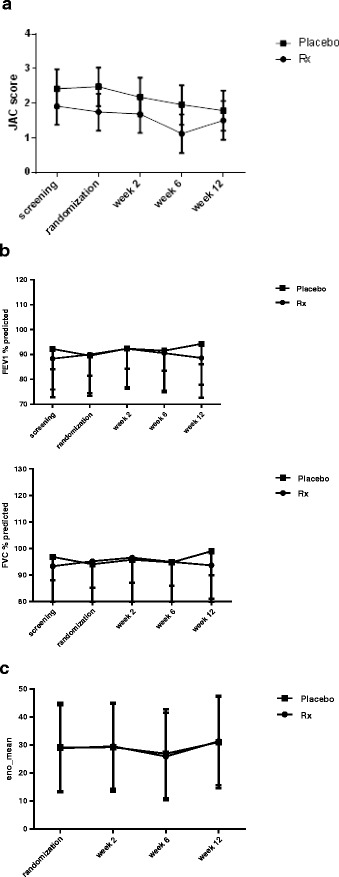
Change in asthma outcomes for participants assigned to placebo and pioglitazone. **a** Juniper asthma control score (*p* = 0.52) (**b**) Lung function (FEV1 and FVC, % predicted) (*p* = 0.36 FEV1 and *p* = 0.15, FVC), and (**c**) exhaled nitric oxide (ppb) (*p* = 0.99). *P*-values are for treatment by time interactions (showing differences between treatment groups), using mixed model repeated measures analysis of variance

There was no significant treatment effect on asthma control as measured by the Juniper asthma control score (Fig. 3a). Neither was there any effect on lung function (Fig. 3b) or exhaled nitric oxide levels (Fig. 3c).

Leptin levels did not change significantly with treatment (placebo 6.7 ± 0.75 at baseline to 6.55 ± 0.5 at week 12 and pioglitazone 6.35 ± 0.6 to 6.55 ± 0.55 ng/ml, *p* = 0.46, repeated measures ANVOA). Adiponectin levels increased slightly more in the pioglitazone group, though this did not reach statistical significance (placebo 409.4 ± 49.6 to 427.7 ± 47.3 ng/ml, pioglitazone 431.6 ± 40.8 to 479.8 ± 45.4 ng/ml. *p* = 0.24 for interaction between treatment and time, repeated measures analysis of variance).

We monitored liver function tests, B-naturetic peptide and complete blood counts in study subjects at each visit; no laboratory abnormalities developed in any of the subjects during study treatment. We asked participants about symptoms and infections at every study visit, and there was no difference in these symptoms or infections between participants assigned to placebo or pioglitazone (Table 3). There was a significant difference in change in weight between the two groups; participants on pioglitazone gained 2.3 kg (mean weight 113.6, CI 94.5-132.7 kg before randomization; 115.9, CI 96.9-135.1 at 12 weeks), whereas participants on placebo lost 3.0 kg over the course of the trial (mean weight 127.5, CI 108.4 – 146.6 kg at randomization; 124.5, CI 105.4 – 143.6 kg at 12 weeks), *p* = 0.04.

**Table 3:** new self-reported symptoms and infections during treatment phase of study

	placebo	Pio	*P*-value*
Fatigue	0	0	-
headache	44	56	0.5
dizziness	11	22	0.5
URI	33	0	0.21
sore throat	22	44	0.62
abdominal pain	44	22	0.62
nausea	11	11	1
diarrhea	33	22	0.5
Skin rash	11	11	1
muscle aches	33	22	0.5
Fluid retention	0	0	-

Values shown are % of patients reporting symptoms

*p value for Fisher’s Exact Test

## Discussion

In this 12 week randomized, placebo-controlled pilot study, we found no difference in pioglitazone versus placebo for the treatment of asthma. Of concern, we found a significant difference in change in weight over the course of the trial: participants on pioglitazone gained significantly more weight than those assigned to placebo. Although only a small pilot trial, these results suggest that systemic pioglitazone is unlikely to be of benefit in the treatment of obese patients with asthma. Although this study suggests that systemic thiazolidinediones are unlikely to be useful for the treatment of asthma because of side-effects, it is possible that an inhaled formulation targeting the airway may obviate some of these side effects.

Animal models of asthma have implicated PPARγ receptors in the airway in the pathogenesis of asthma, and suggested that pioglitazone might be efficacious in the treatment of allergic asthma in lean allergic animal models of asthma [19, 20] A recent single center cross-over study showed that rosiglitazone (another TZD medication, in the same class as pioglitazone) had modest efficacy in reducing the late asthmatic response to allergen challenge in mild-allergic asthmatics [21], and Spears et al. found a significant improvement in lung function in smoking asthmatics treated for 4 weeks with rosiglitazone compared with inhaled corticosteroids [22]. Rosiglitazone has been associated with an increased risk of cardiovascular events [23], and so we chose to study pioglitazone which appears to have a more acceptable safety profile [24].

We stopped our study after recruitment of only 23 participants. During the study, new safety concerns emerged about pioglitazone in the development of bladder cancer [25]. Our power calculations suggested we needed 17 participants per group to detect a statistically significant difference between groups. However, given the new safety concerns, the lack of trend towards efficacy, and possible harm related to weight gain, our results suggest that systemic TZD’s are unlikely to be of benefit in obese asthmatics. Our study does not address whether pioglitazone may be of benefit in lean asthmatics, or if airway targeted therapy might be efficacious in the treatment of obese asthmatics.

Previous studies have implicated adiponectin and leptin in the pathogenesis of asthma in obese individuals. Sood et al. found that high sputum adiponectin was associated with low risk of asthma (obesity is associated with low adiponectin), even after adjustment for markers of adiposity and circulating adiponectin [26]. Shore et al. have shown that increased serum adiponectin attenuates allergic airway responsiveness and inflammation in lean mice [27,28], and Medoff et al. have shown that adiponectin deficiency increases airway eosinophilia [29]. The exact mechanism by which adiponectin may protect against asthma is not known, though adiponectin receptors are expressed by many cells in the lung, and adiponectin may have effects on innate and adaptive immune function [30], smooth muscle [31], bronchial epithelial cells [11] and endothelial function [32]. Leptin, which increases in proportion to obesity, has been associated with asthma in obesity. Visceral fat leptin levels correlate with airway reactivity in obese asthmatics [11], and Shore et al. have found that leptin infusion increases airway reactivity in lean allergen challenged mice [33]. Leptin may have pleotropic effects in the airway: it is involved in neonatal lung development, effects innate and adaptive immunity [34], and parasympathetic signaling in the airway [35]. High leptin and low adiponectin are thought to contribute to asthma in obesity through a number of different pathways.

We do not know why treatment with pioglitazone did not lead to an improvement in asthma in our participants: it is possible that the increase in adiponectin produced by our intervention was not sufficient to have a clinically significant impact on asthma outcomes in this patient population. The dose of pioglitazone used in this study has been reported to increase circulating adiponectin levels, and this strongly correlates with improvement in non-alcoholic fatty liver [12]. In fact pioglitazone, at the doses used in the current trial, has significant efficacy in the treatment of nonalcoholic fatty liver disease, when compared with placebo [12, 36]. However, we did not see a significant increase in adiponectin levels (though there was a numeric increase), and this may be related to the shorter duration of our study than have been used for the treatment of nonalcoholic fatty liver disease. We did not detect any change in leptin levels with pioglitazone treatment: pioglitazone can decrease circulating leptin levels, though this is more variable [37]. It is also possible that this short-term study was not of sufficient duration to significantly alter the pathways that contribute to asthma in obese adults. This may be true if long term obesity significantly affects the mechanical properties of the lung: we have shown that obese asthma is particularly associated with decreased compliance of the lung periphery, and this does not improve with weight loss to the same degree that mechanical changes improve in obese controls without asthma going through bariatric surgery [9]. There are also multiple co-morbidities that we have shown are likely to contribute to asthma control in obesity, such as obstructive sleep apnea and depression [38, 39]; treatment with TZD medication is unlikely to affect these other comorbidities.

We are not aware of any prior studies of TZD’s for the treatment of asthma in obesity. Shore et al. have studied the efficacy of the biguanide metform, an anti-diabetic medication which increases insulin sensitivity through effects on AMP-activated protein kinase: metformin has no effect on airway reactivity or inflammation in response to ozone [40]. Metformin significantly reduced hyperglycemia, but did not affect adiponectin or leptin levels in obese mice, and so this study did not address whether an anti-diabetic medication that could alter these adipokine levels might have efficacy in the treatment of obese asthma.

Weight gain is a recognized side effect of treatment with medications such as pioglitazone. In a 6 month study of pioglitazone (using the identical dose we used in the current trial) and a hypocaloric diet compared with placebo and hypocaloric diet, Belfort et al. found that participants assigned to pioglitazone gained an average of 2.5 kg, and increased body fat by 1.5 %, whereas those on placebo lost a non-significant amount of weight [12]. Participants in this study gained a similar amount of weight over a shorter time period, likely because they were not being treated with a hypocaloric diet. Participants assigned to placebo in our study actually lost weight, we do not know the reason for this, but speculate it may be related to the fact that we discussed the possibility of weight gain during the informed consent period, and all participants were provided with scales to monitor their weight at home during the course of the trial. This may have led to increased awareness of weight, and weight loss in the placebo group.

## Conclusions

In conclusion, although a number of studies suggest there may be a link between adipokines and asthma, the current small study did not show any obvious efficacy for pioglitazone in the treatment of poorly controlled asthma. Of concern, there was a significant weight gain in participants assigned to pioglitazone, which may significantly impact on poor asthma control. These data suggest that there is no role for systemic pioglitazone in the treatment of asthma in obesity; future investigations which uncover the link between obesity and asthma are warranted to direct future intervention trials in this difficult patient population.
